# Top five research priorities in physician-provided pre-hospital critical care – appropriate staffing, training and the effect on outcomes

**DOI:** 10.1186/s13049-020-00724-x

**Published:** 2020-04-30

**Authors:** Marius Rehn, Kristi G. Bache, Hans Morten Lossius, David Lockey

**Affiliations:** 1 Department of Research, The Norwegian Air Ambulance Foundation, Oslo, Norway; 2 Pre-hospital Division, Air Ambulance Department, Oslo University Hospital, Oslo, Norway; 3 Faculty of Health Sciences, University of Stavanger, Stavanger, Norway; 4 Institute of Basic Medical Science, University of Oslo, Oslo, Norway; 5 School of Clinical Sciences, University of Bristol, Bristol, UK

In October 2011, a consensus paper was published in this journal listing the top five topics that an expert group prioritized for pre-hospital critical care research [1]. The journal has revisited these priorities [2] and also published systematic literature reviews summarizing knowledge that has been added since these priorities were defined, on the subjects of point of care ultrasound [2], airway management [3] and the role of dispatch [4]. One topic, appropriate staffing in pre-hospital critical care has remained elusive to investigate.

Despite realizing that the true onset of health compromising acuteness is at the time of injury or disease onset, not when the patient enters the emergency department, the focus remains heavily skewed towards allocating competence and capacity to the receiving hospital. This corresponds to a lack of competence and resources during the pre-hospital phase. This creates a deadly mismatch between a rapidly developing pathophysiological cascade response and our way of organising the chain of emergency medical services (EMS). The more logic continuation in understanding this mismatch would be moving more competence and resources into the pre-hospital setting. Surprisingly, this meets persistent resistance, both within health administrations and within the professional societies. Physician-staffed helicopter EMS (HEMS) have been established throughout Europe to reinforce the regular EMSs with advanced medical interventions and rapid triage and transport to the preferred receiving hospital. The vast majority of physicians staffing these HEMS are specialists in emergency medicine or anaesthesiology, experienced in pre-and in-hospital critical care. In parts of the medical community, the effect on patient outcome of adding HEMS to the regular EMS remain controversial.

Masterson S. et al. have recently published in this journal a systematic review which investigated what clinical crew competencies and qualifications are required for HEMS to provide care that optimizes patient outcomes [3]. They included 38 studies of which the vast majority were of an observational design describing doctor-staffed models. Their conclusions were necessarily very limited due to the poor quality of available studies and possible publication bias weighted towards doctor-staffed models. Nevertheless, they found that HEMS crews provide a wider range of competencies and experience and deliver more key interventions than ground-EMS. Although limited by their observational design, studies comparing HEMS to ground-based EMS also suggested better patient outcomes. Crew configurations are much more complex than they sometimes appear. For example, some studies reasonably choose to compare doctor -paramedic with paramedic- paramedic crews. However, within the professional label of ‘doctor’ or ‘paramedic’ there is significant variability both within counties and internationally. A ‘doctor’ may be an experienced consultant with the full range of pre-hospital critical care skills and sub-speciality accreditation, a trainee with less experience, or a rural general practitioner bringing knowledge and experience, but no critical care interventions to the mission. There are ‘paramedics’, ‘flight paramedics’, ‘critical care paramedics’, ‘advanced paramedics’ and ‘consultant paramedics’ working in pre-hospital care. The training and competences for each role vary significantly nationally and internationally and it can be very difficult to understand what exactly is being delivered to scene in a given study. Given the study heterogeneity, differences in ground EMS competencies and the limited focus on patient outcomes in the literature, Masterson S. et al. were unable to use the evidence to comment on optimal clinical crew competencies and qualifications for HEMS provision.

Despite the declared need for research in this area, there is still little good published evidence which links specific crew configurations to improved patient outcomes. There are many studies which successfully examine the outcome of individual interventions, but the more complex and multifactorial research question of optimal crew configuration is challenging to answer [5]. There are complex intervention methodologies [6] which could address the various components of pre-hospital critical care which can act either independently or interdependently. It is nearly 10 years since the question of how staffing and training influences emergency patient outcomes was added to a priority research question list. It remains unanswered, but is still a relevant research topic. Carefully designed future studies may deliver answers to these questions, but as the review by Masterson et al. demonstrates current published knowledge remains inadequate. It may be that crew configuration should be determined by fully understanding which interventions provide benefit to critically unwell and injured patients and how they can be best provided. With this knowledge the crew configuration will be determined by which crew combination can safely deliver the required interventions in a particular system. This might avoid the difficulties of interpreting the confusing professional labels that currently exist in the literature.
